# Synthesis and Structural Characterization of Substituted 2-Phenacylbenzoxazoles

**DOI:** 10.3390/ijms14034444

**Published:** 2013-02-25

**Authors:** Agnieszka Skotnicka, Erkki Kolehmainen, Przemysław Czeleń, Arto Valkonen, Ryszard Gawinecki

**Affiliations:** 1 Department of Chemistry, University of Technology and Life Sciences, Seminaryjna 3, PL-85-326 Bydgoszcz, Poland; E-Mail: askot@utp.edu.pl; 2 Department of Chemistry, P.O. Box 35, FI-40014 University of Jyväskylä, Finland; E-Mails: erkki.t.kolehmainen@jyu.fi (E.K.); arto.m.valkonen@jyu.fi (A.V.); 3 Department of Physical Chemistry, Collegium Medicum, N. Copernicus University, Kurpińskiego 5, 85-950 Bydgoszcz, Poland; E-Mail: przemekcz@cm.umk.pl

**Keywords:** 2-phenacylbenzoxazole, tautomerism, substituent effect, hydrogen bond, resonance interaction, NMR, quantum-chemical calculations

## Abstract

^1^H and ^13^C NMR spectra of eleven 2-phenacylbenzoxazoles (ketimine form) show that their CDCl_3_-solutions contains also (*Z*)-2-(benzo[*d*]oxazol-2-yl)-1-phenylethenols (enolimine form). Intramolecular hydrogen bonding in the latter tautomer was found to be significantly weaker than that one in respective (*Z*)-2-(2-hydroxy-2-phenylvinyl)pyridines. Integrals of the ^1^H NMR signals were used to evaluate the molar ratio of the tautomers. Strong electron-donating substituents were found to stabilize the ketimine tautomer. p*K*_T_ (negative logarithm of the equilibrium constant, *K*_T_ = [ketimine]/[enolimine]) was found to be linearly dependent on the Hammett substituent constant σ. The results of the MP2 *ab initio* calculations reveal enolimine including an intramolecular OH···N hydrogen bond to be the most stable form both with electron-donor and electron-acceptor substituents. The stability of ketimines is an intermediate of those found for enolimines and enaminones *i.e.*, (*E*)-2-(benzo[*d*]oxazol-2(3*H*)-ylidene)-1-phenylethanones. ^13^C CPMAS NMR spectral data reveal that in the crystalline state the ketimine tautomer is predominant in *p*-NMe_2_ substituted congener. On the other hand, enolimine forms were detected there when the substituent has less electron-donating character or when it is an electron-acceptor by character.

## 1. Introduction

**Scheme 1:**
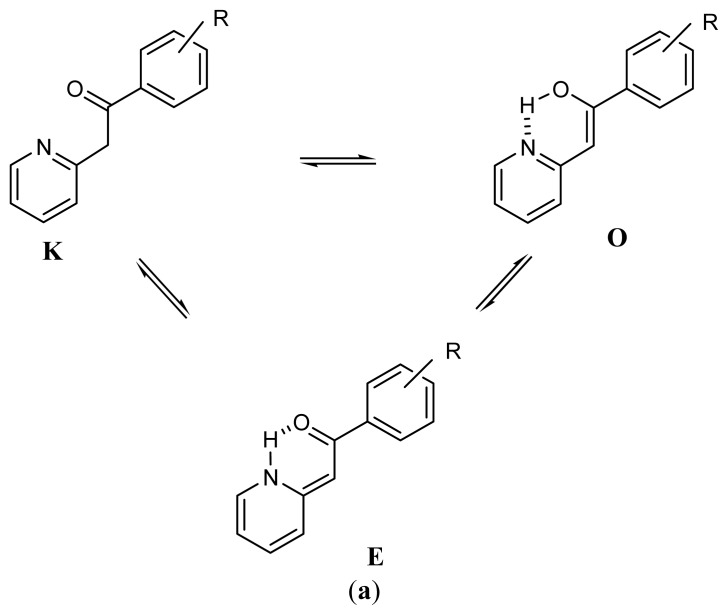

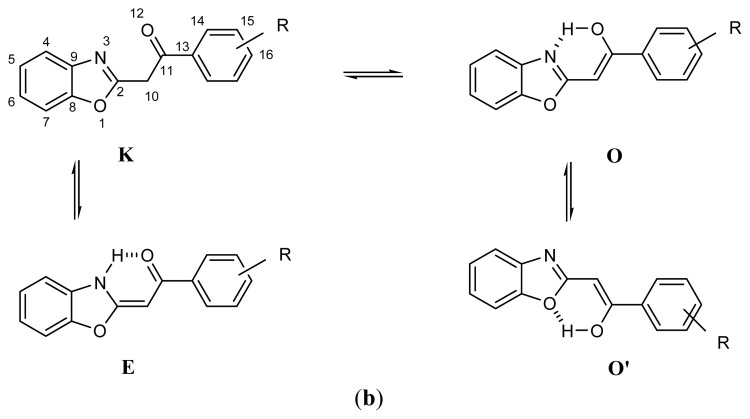
Tautomeric equilibria of 2-phenacylpyridines (**a**) and 2-phenacylbenzoxazoles (**b**).

Acylation of the anion obtained by subtracting one of the methyl protons in 2-methyl(benzo)pyridines affords 2-phenacyl(benzo)pyridines [1–3]. If the pyridine ring is not benzo annulated or if the annulation locates at 4,5-position, their chloroform solutions always contain (Z)-2-(2-hydroxy-2- phenylvinyl)pyridines (enolimine form, Scheme 1) [2,3] in addition to the ketimine form [1–3]. On the other hand, when the pyridine ring is benzo annulated at 3,4- or 5,6-positions or at both of them, the ketimine tautomer is always in equilibrium with (*Z*)-1,2-dihydro-2-benzoyl-methylenepyridines (enaminone form, Scheme 1) [1,3]. Substituents can affect the relative contribution of the respective tautomer even in an almost 100% range [2].

The methyl group in 2-methylbenzoxazole is also susceptible to loss of one proton [4,5]. If this process is followed by acylation of the obtained anion, 2-phenacylbenzoxazoles are the final reaction products [6–8]. Thus, from this point of view, 2-methylbenzoxazole resembles the behavior of 2-methyl(benzo)pyridine [1–3]. The ketimine form, **K**, of 2-phenacylbenzoxazole (Scheme 1) is expected to tautomerize into enolimine, **O**, *i.e.*, (*Z*)-2-(2-hydroxy-2-phenylvinyl)benzoxazole [(*Z*)-2-(benzo[*d*]oxazol-2-yl)-1-phenylethenol)] and enaminone, **E**, *i.e.*, (*E*)-1,2-dihydro-2-benzoylmethylenebenzoxazoles [(*E*)-2-(benzo[*d*]oxazol-2(3*H*)-ylidene)-1-phenylethanone]. Both **O** and **E** tautomers shown in Scheme 1 are stabilized by intramolecular hydrogen bonds.

The UV spectral studies show that in solution 2-phenacylbenzoxazole (see the **K** form in Scheme 1; R = H) is in equilibrium with 2-(benzo[*d*]oxazol-2-yl)-1-phenylethenol (the **O** form in Scheme 1; R = H) [9]. From the ^1^H and ^13^C NMR studies it is known, however, that except the ketimine form (2-phenacylbenzoxazoles), tautomeric enaminones (denoted as E in Scheme 1) are present in CDCl_3_ as well as in CCl_4_ and DMSO-d_6_ solutions [10]. DMSO-d_6_ solution of 2-(p-nitrophenacyl)benzoxazole was also found by ^1^H NMR spectroscopy to contain some amount of an enaminone form [11]. However, other authors using the same technique [12] were not able to see which of the **E** and **O** forms is present in CDCl_3_-solution of 2-phenacylbenzoxazole. Identification of the tautomers was finally cleared out by More O’Ferrall and Murray [13]. These authors have found that NMR signals of H10 at 6.2 ppm, ^13^C10 at 83.7 ppm, ^13^C11 at 166.3 ppm and ^13^C2 at 160.5 ppm (CDCl_3_-solution, *T* = 293–298 K) are characteristic for the enolimine tautomer. The respective chemical shifts for the ketimine form differ significantly from those of enolimine form being: δ(^1^H10) = 4.6 ppm, δ(^13^C10) = 39.7 ppm, δ(^13^C11) = 192.5 ppm and δ(^13^C2) = 165.8 ppm [13]. Similar values of the NMR chemical shifts were found for the respective species present in solutions of 2-phenacyl(benzo)pyridines [1–3].

In order to evaluate the stability of the tautomers, different effects such as steric and electronic (inductive and resonance) interactions have to be considered [1–3]. It is well known that some non-covalent interactions such as inter- and intramolecular hydrogen bonds must also be taken into account when looking for the predominant tautomers [1–3]. Substituents and temperature are other factors that influence the tautomeric equilibria [1–3]. In this study, the tautomeric equilibrium in 2-phenacyl-benzoxazole solutions will be discussed and compared with that of 2-phenacylquinolines from which 2-phenacylbenz-oxazoles differ by an additional heteroatom and the size of the heterocyclic ring.

## 2. Results and Discussion

### 2.1. Synthesis and Identification of Tautomers

Naming of the compounds that undergo tautomerization can be somewhat problematic. Lack of important, e.g., spectroscopic information about the form being detected may result in confusion [3]. Methods as ^13^C CPMAS spectroscopy and X-ray crystallography are helpful in identification of the form present in the crystalline state. The species present in solution were identified (see Discussion), so they are named properly. A complete set of the tautomers that can be present in both states is presented in Scheme 1. Each species is distinguishable by substituent (R = *p*-N(CH_3_)_2_ (**1b**), *p*-OCH_3_ (**2b**), *p*-CH_3_ (**3b**), *m*-CH_3_ (**4b**), H (**5b**), *m*-OCH_3_ (**6b**), *p*-Cl (**7b**), *p*-Br (**8b**), *m*-F (**9b**), *p*-NO_2_ (**10b**), 3,5-(NO_2_)_2_ (**11b**)) and by type of the tautomer. Thus, e.g., 6Ob is the m-OCH_3_ substituted enolimine.

**Scheme 2:**
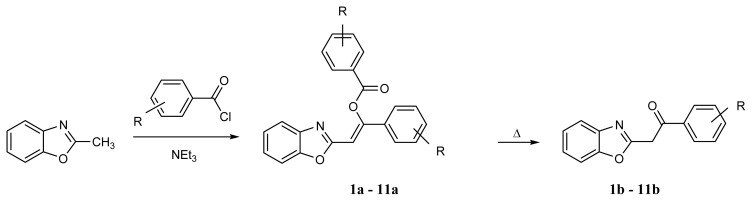
Synthesis of 2-phenacylbenzoxazoles. R = *p*-N(CH_3_)_2_ (**1**), *p*-OCH_3_ (**2**), *p*-CH_3_ (**3**), *m*-CH_3_ (**4**), H (**5**), *m*-OCH_3_ (**6**), *p*-Cl (**7**), *p*-Br (**8**), *m*-F (**9**), *p*-NO_2_ (**10**), 3,5-(NO_2_)_2_ (**11**).

2-Phenacylbenzoxazoles can be obtained in condensation of ortho-aminophenol with 3,3-dialkoxy-, 3,3-dimercapto-1-phenylprop-2-en-1-ones [9,12] or alkyl benzoylacetates [14]. These compounds were also conveniently prepared by subtraction of one of the methyl protons in 2-methylbenzoxazole and treatment of the formed carbanion with an acylating agent such as ester of benzoic acid or benzoyl chloride [6–8,13,15–17] (Scheme 2). The compounds **1b**–**11b** (Scheme 2) discussed in the present paper were obtained most often in the two step synthesis starting from 2-methylbenzoxazole and substituted benzoyl chlorides. The procedure was essentially that used earlier [15].

**Figure 1:**
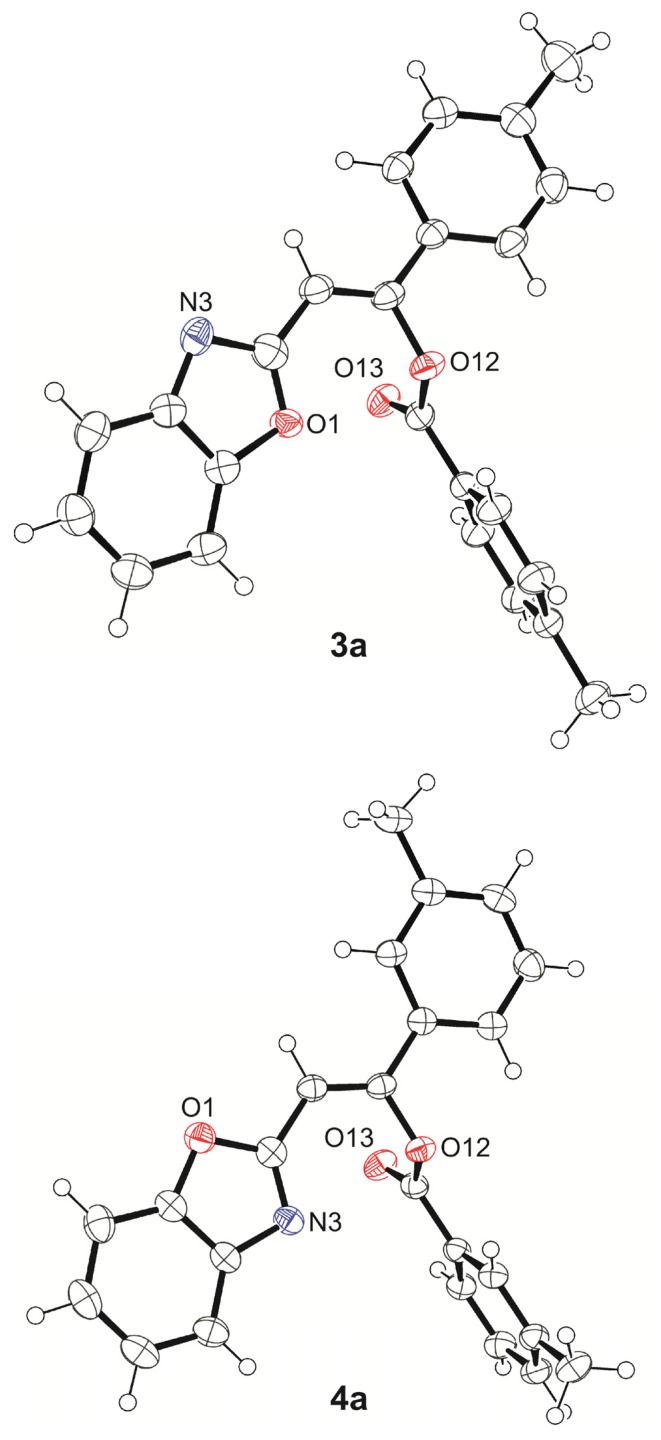
Single crystal thermal ellipsoid structures (ORTEP plots) of **3a** and **4a**. The positional disorders in methyl groups (H-atoms) and in benzoxazole moiety of **3a** were removed for clarity.

Since treatment of 2-methylbenzheteroazoles with benzoyl chloride and triethylamine may afford a variety of products [16,18,19], the nature of the intermediate products can be doubtful. When reaction takes place at 100 °C, the 2-methyl group in 2-methylbenzoxazole undergoes double benzoylation, *i.e.*, it is transformed to –CH=C(Ph)–COOPh. According to Ciurdaru and Ciuciu [15] 2-(benzo[*d*]oxazol-2-yl)-1-phenylvinyl benzoate, **5a**, is the reaction product (identification was based only on its IR spectra and reactivity [15]). The X-ray structures of **3a** and **4a** (Figure 1) show without any doubt that the intermediate products are really (*Z*)-2-(benzo[*d*]oxazol-2-yl)-1-phenylvinyl benzoates. The bond lengths and angles as well as torsion angle for 3a and 4a are available in Supplementary Materials Deposit. It is noteworthy that conformations of these two compounds in crystals can be different, as observed from Figure 1, where the benzoyl moiety is shown to locate close to O1 (**3a**) and to N3 (**4a**). However, the benzoxazole moiety in **3a** shows positional disorder (1:1) and in half of the molecules in crystal state have the similar conformation to **4a** (benzoyl close to N3).

Intermediate products, *i.e.*, 2-(benzo[*d*]oxazol-2-yl)-1-phenylvinyl benzoates [(*E*)-phenyl 3-(benzo[*d*]oxazol-2-yl)-2-phenylacrylates], were isolated from the reaction mixture and characterized. Their synthetic and physical data as well as selected ^1^H and ^13^C NMR chemical shifts are presented in Tables 1 and 2, respectively. ^15^N NMR chemical shifts for selected 2-(benzo[*d*]oxazol-2-yl)-1-phenylvinyl benzoates show that the respective chemical shifts do not vary significantly being −131.8 ppm and −131.2 ppm for **4a** and **6a** as examples. Non purified intermediate products **1a**–**11a** were always used in the next synthetic step.

By refluxing their morpholine solutions, **1a**–**11a** can be easily transformed into 2-phenacylbenzoxazoles **1b**–**11b** (method B). These products can be also obtained in one step starting from 2-methylbenzoxazole and substituted ethyl benzoate (the methyl proton was subtracted by sodium hydride) [8] (method A). Synthetic and physical data for 2-phenacylbenzoxazoles prepared by these two procedures are shown in Table 3.

It is easy to distinguish 2-phenacylbenzoxazole (no ^1^H NMR signals above 10 ppm) from its **O** and **E** tautomers (these species contain the N**H** and O**H** protons). Since the chemical shifts of such acidic protons are comparable [1–3], identification of the **O** and **E** forms cannot base on the shifts of these signals. ^1^H NMR chemical shift data in Table 2 show that hydroxy protons, H10, in (*Z*)-2-(2- hydroxy-2-phenylvinyl)benzoxazoles ((*Z*)-2-(benzo[*d*]oxazol-2-yl)-1-phenylethenols) (enolimines, **O**) (Scheme 1) are much more shielded (less acidic) than these in (*Z*)-2-(2-hydroxy-2-phenylvinyl)-pyridines and their benzo derivatives (δ(O**H**) = 14.41–15.58 ppm) [2,3]. Thus, intramolecular hydrogen bond in the former compounds is significantly weaker than this in the respective pyridine derivatives.

NMR signals of other protons, e.g., H10, seem also helpful in distinguishing between different tautomeric forms present in solution. Although signal of H10 in the spectra of 2-phenacyl(benzo)pyridines (**K**) was observed in the 4.5–5.5 ppm range, for the other tautomers its positions were comparable: 5.9–6.2 ppm (**O**) and 6.0–6.8 ppm (**E**) [1–3]. Thus, comparison with the respective data in Table 4 does not reveal whether another tautomer in CDCl_3_-solution is **O** or **E**. The ^13^C10 chemical shifts are also comparable for some of these compounds and their tautomeric forms: δ(^13^C10) = 46.5–49.4 ppm (**K**), 83.6–90.3 ppm (**O**) and 91.1–96.8 ppm (**E**) [1–3]. On the other hand, chemical shift of ^13^C11 are significantly different for every individual tautomer: δ(^13^C11) = 194.2–196.9 ppm (**K**), 181.7–187.1 ppm (**O**) and 160.0–163.4 ppm (**E**) [1–3]. The values of δ(^13^C11) = *ca* 166 ppm (Table 4) do not reveal unequivocally whether there is the **O** or **E** tautomer in CDCl_3_-solution. The ^15^N chemical shifts of respective (benzo)pyridines seem to be the most useful parameter to distinguish between these three tautomers: δ(^15^N3) = 68.2–78.4 ppm, 226.2–261.4 ppm and 105.6–125.8 ppm for **K**, **O** and **E** forms, respectively [1–3,21,22]. Since the pyridine and oxazole heterocycles are different, however, the direct comparison of their ^15^N NMR data is not reasonable.

The integrals of H10 signals were used to calculate the molar ratio of different forms present in solution (Table 5). Accuracy of these data was supported by evaluation based on signal intensities of the substituent protons and the literature data. In many cases, mainly π-electron delocalization was found to be responsible for tautomeric preferences [23] but also other effects such as the strength of the intramolecular hydrogen bond should to be taken into account.

### 2.2. Substituent Effect on the Tautomeric Equilibrium

**Scheme 3:**
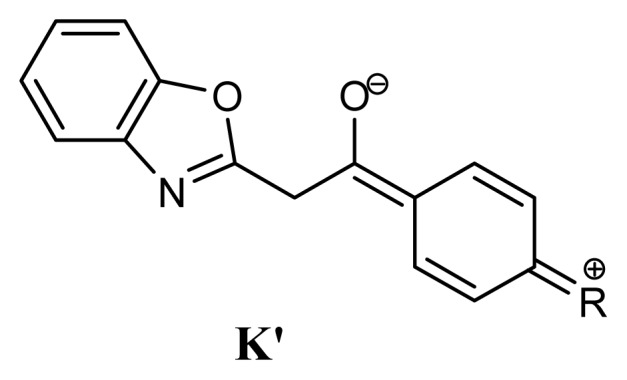
Resonance structure stabilizing the 2-phenacylbenzoxazole tautomer K by electron-donating substituents.

As can be seen in Table 5, tautomeric ratio in solutions of **1b**–**11b** depends strongly on the substituent. Electron-acceptor substituents increase the acidic character of methylene protons in the **K** forms (electron-donating substituents in the phenacyl part of the molecule favor the **K** form, Scheme 3). As a consequence, their transfer to the *aza* atom in these compounds is easy.

**Scheme 4:**
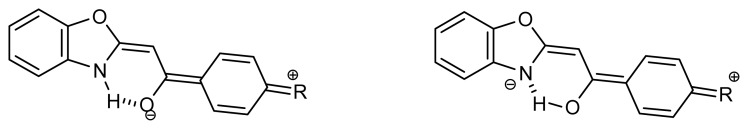
Resonance structures showing lack of stabilization of the tautomers **E** and **O** by electron-donating substituents.

Notwithstanding the **E** and **O** forms are stabilized by the intramolecular hydrogen bonds, those carrying electron-donating substituents are not stabilized because neither the oxazole nor benzene rings in their molecules are aromatic (Scheme 4).

**Figure 2:**
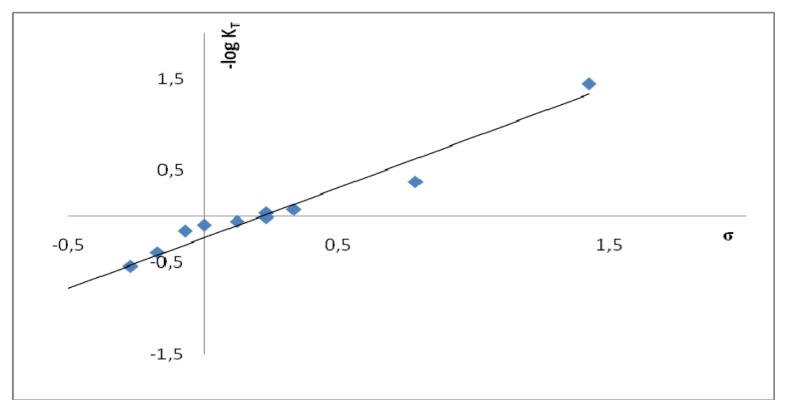
Plot of −log***K***_T_*vs.* Hammett substituent constant σ for **1b**–**11b**.

Data in Table 5 and our earlier results [2] reveal that the substituent effect on the content of 2-phenacylbenzoxazoles and 2-phenacylpyridines (ketimine form) is of the same type. Although the dependence between p*K*_T_ and Hammett substituent constant σ [24] has a linear character both for benzoxazole (p*K*_T_ = 1.10σ − 0.23, *R* = 0.984, Figure 2) and pyridine (p*K*_T_ = −1.53σ + 0.27 [2]) derivatives, changes in [**K**]’s for these two species are not parallel. Thus, nevertheless [**K**] for 2-(p-dimethylaminophenacyl) derivatives of benzoxazole and pyridine are comparable (94.5% (Table 5) and 95.7% [2], respectively), contribution of the ketimine form in solutions of the 2-(p-nitrophenacyl) derivatives are significantly different (29.5% (Table 5) and 7.8% [2], respectively). Some extraordinary stabilization of the **O** tautomer, or extraordinary destabilization of the **K** tautomer by the substituent can be responsible for this behavior. Since both in 2-phenacylbenzoxazoles and 2-phenacylquinolines the ring that contains a nitrogen atom is benzo annulated, these two series are worthy to be compared. Thus, for the later series p*K*_T_ was also found to be linearly dependent on the substituent constant [1] (p*K*_T_ = −1.10σ − 1.30). One should keep in mind that in both series the tautomeric mixture contains **K** and **O**. On the other hand, there is **E** (instead of **O**) present in chloroform solution of quinoline derivatives. The amount of **K** in solutions of all three series (2-phenacyl derivatives of benzoxazole, pyridine and quinoline) are equal to 65%, 88% and 34%, respectively (*p*-NO_2_ and *p*-N(CH_3_)_2_ are the most extreme substituents studied). Thus, the substituent effect is the most significant in the pyridine series.

The above discussion shows that benzoxazoles resemble the pyridine derivatives because their tautomeric mixture contains **K** and **O**. Moreover, [**K**] vary in a wide range for these series (Table 5). On the other hand, benzoxazoles resemble also the quinoline derivatives: these compounds are benzo annulated oxazoles and pyridines, respectively. Except the common **K**, another tautomer present in their chloroform solutions is **O** and **E**, respectively. However, the contributions of **K** in these two series differ significantly. Content of the tautomeric mixtures for all three series (benzoxazoles, pyridines and quinolines) is really sensitive to the substituent present in the molecule. Although the dependence between pK_T_ and substituent constant is always linear by character, pK_T_ = ρσ + const, changes in [**K**] in these series are not parallel.

Solid state ^13^C CPMAS NMR spectra are known to be very helpful in identification of the form present in the crystalline state. Thus, **1K** was detected for the *p*-NMe_2_ substitution (Table 4). On the other hand, **3O**, **9O** and **10O** are present in their crystals (substituent present in their molecules has less electron-donor character (*p*-Me) or it is of electron-acceptor type (*m*-F, *p*-NO_2_)). Thus, substituent effect on the tautomer present in the crystals of oxazoles studied is the same as in the respective pyridine derivatives [2].

### 2.3. Quantum-Chemical Calculations

Some theoretical calculations were performed for the compounds studied in order to support the obtained experimental data. MP2 is known to be the most accurate and effective *ab initio* method for studying the medium size molecules involving hydrogen bonds [25]. Tautomers studied in the present paper meet such the requirements. This procedure includes electron correlation, so the calculated and experimental data are expected to be comparable [26]. Some optimized bond lengths and bond and dihedral angles in the molecules of 2-phenacylbenzoxazoles and their tautomers are presented in Table 6. Judging from the length of hydrogen bond, it seems to be stronger in enolimines than in enaminones. Of the OH···N and NH···O interactions, the former is stronger. Intramolecular hydrogen bond is especially weak in **5O′**. C14C13C11O12 and C18C13C11O12 dihedral angles prove H10 and H18 to interact sterically. Repulsion between these hydrogen atoms is rather weak in the **K** forms. Comparison of H10···H18 distances (Table 6) enables evaluation of the strength of the interaction in other tautomers. Since the **E** molecules are most crowded, this form is unstable.

The calculated energies of different tautomers (Table 7) prove the **O** form including the OH···N hydrogen bond to be the most stable (both electron-donor and electron-acceptor substituents follow this rule). **K** forms are less stable than enolimines (the more electron-donor is the substituent, the more stable is the ketimine form). Calculations for R = H show that the **O′** form is much more unstable than the **O** form. As this can be seen in Scheme 1, the six-membered pseudo ring including the OH···O system in **O′** is less aromatic than the respective pseudo ring including the OH···N moiety in **O**. The strong resonance assisted hydrogen bonds (RAHB), such as this present in the molecule of the later tautomer, are well known [27–30]. The less stable tautomer is always **E** (both electron-donor and electron-acceptor substituents follow this rule).

Since the most stable tautomers, *i.e.*, **O** and **K**, were really found to be present in chloroform solution, one may see that solvent does not affect tautomeric preferences.

## 3. Conclusions

In CDCl_3_ solution 2-phenacylbenzoxazoles (ketimine tautomeric form) are in equilibrium with (*Z*)-2-(benzo[*d*]oxazol-2-yl)-1-phenylethenols (enolimine form). (*E*)-2-(benzo[*d*]oxazol-2(3*H*)- ylidene)-1-phenylethanones (enaminone) were not detected. Thus, 2-phenacylbenzoxazoles resemble the respective pyridine derivatives. Intramolecular hydrogen bond in enolimines is always significantly weaker than this in the respective (*Z*)-2-(2-hydroxy-2-phenylvinyl)pyridines. The molar ratio of different forms in solution (based on the integrals of ^1^H NMR signals) depends on substituent. Electron-withdrawing substituents increase the acidic character of methylene protons in the ketimine forms. In consequence, transfer of such a proton to the carbonyl oxygen is very easy in these compounds. On the other hand, electron-donating substituents in the phenacyl part of the molecule favor the ketimine form. Substituent effect on the content of 2-phenacylbenzoxazoles and 2-phenacylpyridines (ketimine form) is of the same type. Although the dependence between p*K*_T_ (*K*_T_ = [**K**]/[**O**]) and Hammett substituent constant σ has a linear character both for benzoxazole and pyridine derivatives, variations in [**K**]’s for these two series are not parallel. Judging from the MP2 optimized lengths of the hydrogen bonds, it seems to be stronger in enolimines than in enaminones. The calculated energies of different tautomers prove the enolimine form including the OH···N hydrogen bond to be the most stable (both electron-donor and electron-acceptor substituents follow this rule). The ketimine tautomers are always less stable than enolimines. The most labile tautomer is always enaminone. Ketimine tautomer was detected for *p*-NMe_2_ substituted congener by the solid state ^13^C CPMAS NMR. On the other hand, enolimine forms are present in their crystals when the substituent present in their molecules has less electron-donor character (*p*-Me) or when it is of electron-acceptor type (*m*-F, *p*-NO_2_).

## 4. Experimental and Computational

Melting points were measured on a Boetius table and are uncorrected. Satisfactory elemental analyses (±0.30% for C, H and N) were obtained for all compounds prepared. All measurements were performed in 24 h after preparing their chloroform solutions (extra signals seen in the NMR spectra recorded two-three weeks later proves slow decomposition of some 2-phenacylbenzoxazoles takes place in this solvent).

### 4.1. X-Ray Crystallography

The single crystals suitable for structure determination by X-ray diffraction experiments were obtained by very slow evaporation of the NMR samples. The structural data were collected at 123.0 ± 0.1 K with Agilent SuperNova dual wavelength diffractometer, using micro-focus X-ray source and multilayer optics monochromatized CuKα radiation (λ = 1.54184 Å). The data collection, reduction and multi-scan absorption correction were made by program CrysAlisPro [31]. The structures were solved by direct methods, using SIR-2004 [32], and refined on *F*_2_ using SHELXL-97 [33]. The hydrogen atoms were treated with riding model. The thermal ellipsoid (50% probability) diagrams were drawn with ORTEP-3 [34]. CCDC-922098 (**3a**) and CCDC-922097 (**4a**) contain the supplementary crystallographic data for this paper. These data can be obtained free of charge from the Cambridge Crystallographic Data Centre via http://www.ccdc.cam.ac.uk/data_request/cif. Crystal data and structure refinement parameters, atomic coordinates, isotropic and anisotropic displacement parameters as well as geometric parameters for **3a** and **4a** are available in Supplementary Materials Deposit.

### 4.2. NMR Spectroscopy

The ^1^H, ^13^C and PFG ^1^H,^13^C HMQC and HMBC spectra were recorded for dilute CDCl_3_ solution in a 5 mm sample tube at 303 K on a Bruker Avance DRX 500 spectrometer equipped with an inverse detection probehead and z-gradient accessory working at 500.13 MHz and 125.77 MHz, respectively.

In ^1^H NMR experiments, the number of data points was 64 K giving a spectral resolution of 0.05 Hz, the number of scans was 8 and the flip angle 30°. An exponential window function of the spectral resolution was used prior to FT. The ^1^H chemical shifts are referenced to the signal of internal TMS at δ = 0.00 ppm.

In ^13^C experiments the number of data points was 32 K giving a spectral resolution of 0.5 Hz, the number of scans vary between 1000 and 10,000 depending on case and flip angle was 30°. A composite pulse decoupling, Waltz-16, was used to remove proton couplings. An exponential window function of the spectral resolution was used prior to FT. The ^13^C chemical shifts are referenced to the signal of internal TMS at δ = 0.00 ppm.

The number of data points in PFG ^1^H, ^13^C HMQC and HMBC measurements were 1024 (*f*_2_) × 256 (*f*_1_). This matrix was zero filled to 2048 × 512 and apodized by a shifted sine bell window function along both axes prior to FT.

In PFG ^1^H, ^15^N HMBC experiments a 100 ms delay was used for evolution of long-range couplings. The number of data points was 1024 (*f*_2_) × 512 (*f*_1_ = ^15^N). This matrix was zero filled to 2048 × 1024 and apodized by a shifted sine bell window function along both axes prior to FT. ^15^N NMR chemical shifts are referenced to the signal of an external CH_3_NO_2_ (δ = 0.0 ppm) in a 1 mm diameter capillary inserted coaxially in the 5 mm diameter NMR tube.

The solid state ^13^C CPMAS NMR spectra were recorded on a Bruker Avance 400 FT NMR spectrometer using samples packed in 4.0 mm o.d. zirconia rotors. The samples were spun at 10 KHz rate and >1000 transients were accumulated. The FIDs are apodized by 10 Hz exponential window before FT. The shifts are referenced to the C=O signal of glycine standard at δ = 176.03 ppm. All acquisition and processing parameters are available from E.K. on request.

### 4.3. Syntheses of 2-Phenacylbenzoxazoles

#### 4.3.1. Method A

The known procedure [8] was slightly modified to obtain **1b**, **2b** and **9b**. Thus, mixture of 2-methylobenzoxazole (2.66 g, 0.02 mol), sodium hydride (60% suspension of in mineral oil, 5.32 g, 0.10 mol) and ethyl benzoate (0.02 mol) in dry toluene (33 mL) was heated at 70 °C for 12 h. Concentrated hydrochloric acid (3 mL) was then added carefully to the stirred and cooled reaction mixture (its temperature should not exceed 5 °C when destroying the residual sodium hydride). This step was followed by the consecutive addition of 20% hydrochloride acid (15 mL) and water (75 mL). The precipitated solid was recrystallized from ethanol. For the reaction yields and melting points of the products see Table 3.

#### 4.3.2. Method B

Benzoyl chloride (0.04 mol) was added in one portion to the stirred solution of 2-methylbenz-oxazole (2.66 g, 0.02 mol) and trimethylamine (11.2 mL, 8.1 g, 0.08 mol) in diglyme (8 mL). Content of the reaction vessel was heated for 1 h at the boiling water bath. Dropwise addition of water (60 mL) to the stirred cold reaction mixture resulted in precipitation of crude 2-(benzo[*d*]oxazol-2-yl)-1-phenyl-vinyl benzoates **3a**–**8a** and **10a**. Analytical samples of these compounds were prepared by repeated crystallization from ethanol. For the reaction yields and melting points of the products see Table 1.

Solution of the crude **3a**–**8a** and **10a** (0.006 mol) and morpholine (1.6 mL, 1,57 g, 0.018 mol) in methanol (9 mL) was refluxed with stirring for 10 min. Water (9 mL) was then added to the boiling reaction mixture which was then cooled down to start precipitation. Crystallization of the collected solid from methanol affords pure 2-phenacylbenzoxazoles **3b**–**8b** and **10b**. For the reaction yields and melting points of the products see Table 3.

**3a**: C_23_H_19_NO_3_ (357.39): calcd. C 77.29, H 5.36, N 3.92; found C 77.04, H 5.47, N 4.08.
**4a**: C_23_H_19_NO_3_ (357.39): calcd. C 77.29, H 5.36, N 3.92; found C 77.09, H 5.11, N 4.06.
**6a**: C_23_H_19_NO_5_ (389.39): calcd. C 70.94, H 4.92, N 3.60; found C 70.69, H 4.68, N 3.69.
**7a**: C_21_H_13_Cl_2_NO_3_ (398.23): calcd. C 63.33, H 3.29, N 3.52; found C 63.12, H 3.47, N 3.39.
**8a**: C_21_H_13_Br_2_NO_3_ (487.15): calcd. C 51.77, H 2.69, N 2.88; found C 51.85, H 2.82, N 3.01.
**10a**: C_21_H_13_N_3_O_7_ (419.33): calcd. C 60.15, H 3.12, N 10.02; found C 59.90, H 3.01, N 9.81.
**1b**: C_17_H_16_N_2_O_2_ (280.32): calcd. C 72.83, H 5.75, N 10.00; found C 72.91, H 5.55, N 9.79.
**9b**: C_15_H_10_FNO_2_ (255.24): calcd. C 70.58, H 3.95, N 5.49; found C 70.73, H 4.06, N 5.70.
**11b**: C_15_H_9_N_3_O_6_ (327.24): calcd. C 55.05, H 2.77, N 12.84; found C 54.95, H 3.01, N 12.62.

### 4.4. Quantum-Chemical Calculations

Geometries for the isolated molecules (vacuum) of the tautomers were optimized using the second order Möller-Plesset method (MP2) [35,36]. Computations were carried out utilizing the augmented correlation-consistent basis set with polarized valence of double-zeta quality (aug-cc-pvdz) [37,38]. All calculations were realized with use of Gaussian 09 package [39].

## Supplementary Information



## Figures and Tables

**Table 1:** Reaction yields and melting points of compounds **3a**–**8a** and **10a**.

No	Yield (%)	mp (°C)
**3a**	35	92–93
**4a**	20	91–92
**5a**	52	104–106 (109 [15], 97–98 [20])
**6a**	30	111–112
**7a**	32	106–107
**8a**	75	175–177
**10a**	63	226–227

**Table 2:** Selected ^1^H and ^13^C NMR chemical shifts (δ from TMS) for 0.1–0.2 M solutions of 2-(benzo[*d*]oxazol-2-yl)-1-phenylvinyl benzoates **3a**–**8a** and **10a** in CDCl_3_ at 303 K.

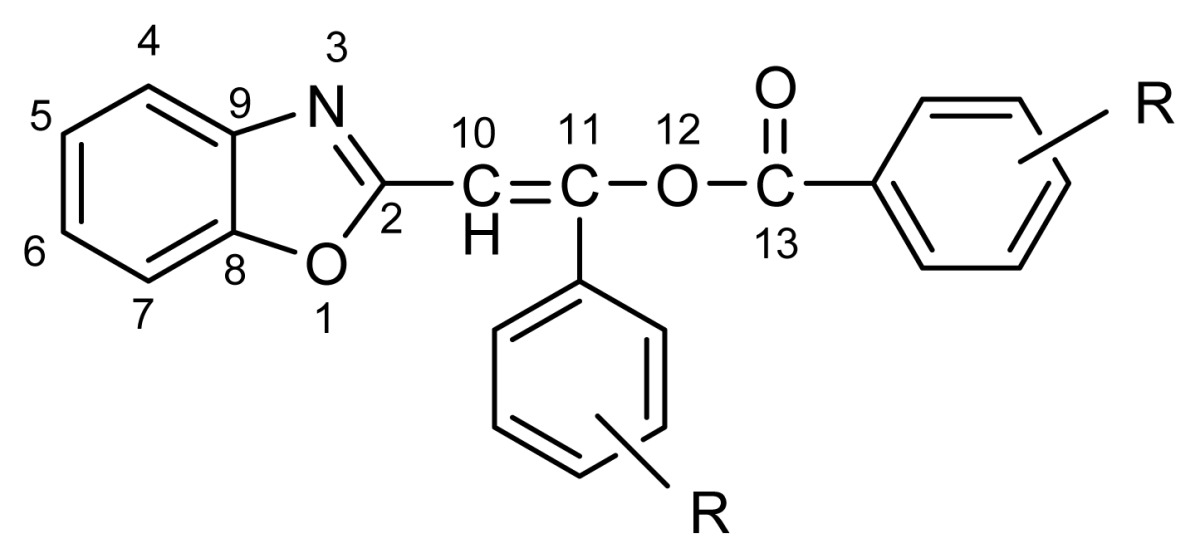					

Compound	H10	C2	C10	C11	C13
**3a**	6.98	164.55	102.39	154.47	160.14
**4a**	7.03	164.70	103.12	154.49	159.96
**5a**	7.26	164.51	103.31	154.20	159.83
**6a**	7.03	164.38	103.61	153.99	159.99
**7a**	7.02	163.28	104.51	152.45	160.86
**8a**	7.00	163.77	103.81	152.94	161.45
**10a**	7.17	162.61	106.75	151.23	158.35

**Table 3:** Reaction yields and melting points of compounds **1b**–**11b**.

Compound	Method a	Yield (%)	mp (°C)
1b	A	73	197–198
2b	A	62	106–108 (107.5–108.5) [8]
3b	B	65.5	96–98 (97.5–98.5) [8]
4b	B	84	66–68 (67–68) [8]
5b	B	59	94–96 (93.5–94.5) [8] (97–98) [12] (88–88.5) [6] (88–88.5) [9] (90–91) [17] (88) [7] 87–89 [20]
6b	B	61.5	58–60 (59.5–60) [8]
7b	B	87	168–170 (168.5–170) [8]
8b	B	83	167–169 (167) [7]
9b	B	50.5	101–102.5
10b	B	99	233–235 b 248–249 [11]
11b	B	72	156.5–158

a Method A: consecutive treatment of 2-methylbenzoxazole by sodium hydride and substituted ethyl benzoate; Method B: decomposition of the product of reaction of 2-methylbenzoxazole with benzoyl chloride.

b Some polymorphic processes take place in the range 200–230 °C.

**Table 4:** Selected NMR chemical shifts for ^1^H from TMS, ^13^C from TMS, ^15^N from ext. CH_3_NO_2_ (regular characters), and ^13^C CPMAS from glycine (italics) of 2-phenacylbenzoxazoles (**K**) and (*Z*)-2-(benzo[*d*]oxazol-2-yl)-1-phenylethenols (**O**).

Tautomer a	OH b	H10 c	C10	C11	N3
**1K**	-	4.54	39.08	190.00	−135.6 d
	*-*	*-*	38.76	100.72	-
**1O**	e	6.05	80.35	166.61	−168.3 e
**2K**	-	4.61	39.39	190.81	−134.9
**2O**	12.6	6.11	82.19	166.35	−166.2
**3K**	-	4.61	39.50	191.95	−134.7
**3O**	12.5	6.17	82.97	166.44	−164.9
	*-*	*-*	82.22	166.49	*-*
**4K**	-	4.62	39.58	192.56	−134.6
**4O**	12.4	6.20	83.61	166.48	−164.2
**5K**	-	4.64	39.59 f	192.36 g	−134.4
**5O**	12.5	6.21	83.69 h	166.29 i	−164.0
**6K**	-	4.62	39.67	192.20	−134.4
**6O**	12.5	6.20	83.91	166.08	−164.0
**7K**	-	4.60	39.63	191.18	−134.1
**7O**	12.7	6.18	83.93	165.48	−164.0
**8K**	-	4.59	39.61	191.39	−134.3
**8O**	12.7	6.19	83.97	165.46	−164.0
**9K**	-	4.61	39.71	191.20	−134.0
**9O**	12.7	6.20	84.44	165.37	−163.3
	*-*	*-*	83.05	162.42	*-*
**10K**	-	4.67	j	j	j
**10O**	12.8	6.34	86.47	164.80	−160.6
	*-*	*-*	84.70	163.16	*-*
**11K**	-	4.74	j	j	j
**11O**	k	6.43	86.47	165.29	−163.9 l

a Recorded for 0.1–0.2 M solutions in CDCl_3_ at 303 K;

b Very broad singlet;

c Singlet.

d δ[^15^**N**(CH_3_)_2_] = −323.9 ppm (form **K**);

e Due to low contribution of the **O** form this signal is not observed (or it is very weak);

f 39.7 ppm [13];

g 192.5 ppm [13];

h 83.7 ppm [13];

i 16.3 ppm [13];

j Due to low contribution of the **K** form this signal is not observed;

k Signal is not seen;

l Two signals at −19.9 ppm and −18.8 ppm were observed for the substituent nitrogen.

**Table 5:** Content of the **K** form (%) (in CDCl_3_ at 303 K), [**O**] (%) = 100 − [**K**] (%).

	[K] (%) a
1	94.5 (87.0) b
2	77.5 (83.0) c
3	71.0 (72.0) c; 56 d,e[10]
4	59.0 (57.5) c
5	55.5; 50 d,e[10,13]; 51.5 [20]; 20 d,f[12]
6	53.0 (50.0) c
7	48.0; 33 d,e[10]
8	50.5
9	45.5
10	29.5
11	3.5

a Based on integrals of the H10 signals (present paper);

b Since the ^1^H NMR chemical shifts for various tautomers differ insignificantly (see Table 4), contributions based on integrals of the N(CH_3_)_2_ protons are not precise;

c Values in parentheses are based on integrals of the substituent protons;

d Literature data for chloroform solutions at 298 K;

e Only K and O tautomers were detected in CDCl_3_;

f There are three different forms in CDCl_3_: [K] + [O] + [E] = 20% + 37% + 43% [12].

**Table 6:** Optimized (MP2/aug-cc-pvdz) bond lengths (Å) and bond and dihedral angles (deg) for 2-phenacylbenzoxazoles and their tautomers.

	O12-H12 or N3-H3	H12···N3 or H12···O1	H10···H18	C14C13C11O12 C18C13C11O12
**1K**	-	-	2.34 2.38 b	−179.97 −0.20
**1O**^a^	1.00	1.76	2.14	164.24 −15.76
**1E**	1.03	1.79	2.04	173.70 −5.56
**5K**	-	-	2.34 2.36 b	179.48 −0.41
**5O**^a^	1.00	1.76	2.20	157.31 −22.36
**5O′**^a^	0.98	1.87	2.19	154.73 −24.91
**5E**	1.04	1.80	2.14	158.62 −20.62
**10K**	-	-	2.35 2.37 b	179.56 −0.33
**10O**^a^	1.00	1.75	2.14	163.19 −16.25
**10E**	1.03	1.80	2.13	160.00 −18.75

a Forms O and O′ include the OH···N and OH···O intramolecular hydrogen bonds, respectively;

b Distances to H18 from two different H10.

**Table 7:** MP2 calculated relative energies (kJ mol^−1^) of different tautomers.

Name	Name
1K	4.18
1O	0.00 a
1E	25.08
5K	12.54
5O	0.00 b
5O′	25.08
5E	29.26
10K	16.72
10O	0.00 c
10E	25.08

a Absolute energy: −914.853 Hartree;

b Absolute energy: −781.273 Hartree;

c Absolute energy: −985.352 Hartree.
