# Supplementary material for: Synthesis and Structural Characterization of Substituted 2-Phenacylbenzoxazoles
Source: Int J Mol Sci. 2013 Feb 25;14(3):4444–60. doi: 10.3390/ijms14034444 (PMC3634406; doi:10.3390/ijms14034444)
Supplement: Supplementary file 1 [file ijms-14-04444-s001.doc]

**Supplementary Materials** **Deposit**

**Table S1.** Crystal data and structure refinement for **3a**.

| CCDC code | 922097 |  |
| --- | --- | --- |
| Empirical formula | C_24_H_19_NO_3_ |  |
| Formula weight | 369.40 |  |
| Temperature | 123(2) K |  |
| Wavelength | 1.54184 Å |  |
| Crystal system | Triclinic |  |
| Space group | *P*-1 |  |
| Unit cell dimensions | a = 9.0668(4) Å | α = 71.159(4)° |
|  | b = 10.6056(5) Å | β= 77.492(4)° |
|  | c = 10.7678(5) Å | γ = 82.891(4)° |
| Volume | 955.06(8) Å^3^ |  |
| Z | 2 |  |
| Density (calculated) | 1.285 Mg/m^3^ |  |
| Absorption coefficient | 0.682 mm^−1^ |  |
| F(000) | 388 |  |
| Crystal size | 0.20 × 0.12 × 0.03 mm^3^ |  |
| Theta range for data collection | 4.41 to 69.98° |  |
| Index ranges | −11 ≤ h ≤ 11, −12 ≤ k ≤ 12, −13 ≤ l ≤ 13 |  |
| Reflections collected | 16636 |  |
| Independent reflections | 3597 [R(int) = 0.0497] |  |
| Completeness to theta = 69.98° | 99.9% |  |
| Absorption correction | Semi-empirical from equivalents |  |
| Max. and min. transmission | 0.9798 and 0.8757 |  |
| Refinement method | Full-matrix least-squares on F^2^ |  |
| Data / restraints / parameters | 3597 / 40 / 271 |  |
| Goodness-of-fit on F^2^ | 1.048 |  |
| Final R indices [I > 2sigma(I)] | R1 = 0.0384, wR2 = 0.0938 |  |
| R indices (all data) | R1 = 0.0486, wR2 = 0.1020 |  |
| Largest diff. peak and hole | 0.189 and −0.201 e.Å^−3^ |  |

**Table S2.** Atomic coordinates (×10^4^) and equivalent isotropic displacement parameters
(Å^2^ × 10^3^) for **3a**. U(eq) is defined as one third of the trace of the orthogonalized U^ij^ tensor.

|  | **x** | **y** | **z** | **U(eq)** |
| --- | --- | --- | --- | --- |
| O(12) | 6932(1) | 787(1) | 3424(1) | 32(1) |
| O(13) | 9211(1) | 279(1) | 2304(1) | 34(1) |
| O(1) | 8106(19) | 3202(12) | 2847(14) | 30(2) |
| N(3) | 8450(30) | 3834(17) | 4571(19) | 36(2) |
| N(1) | 8060(20) | 3319(17) | 2673(18) | 36(2) |
| O(3) | 8570(20) | 3634(13) | 4523(14) | 32(2) |
| C(2) | 8185(2) | 2807(2) | 4056(2) | 39(1) |
| C(4) | 9078(2) | 6129(2) | 3373(2) | 41(1) |
| C(5) | 9219(2) | 7068(2) | 2117(2) | 42(1) |
| C(6) | 8962(2) | 6772(2) | 1013(2) | 43(1) |
| C(7) | 8540(2) | 5520(2) | 1120(2) | 39(1) |
| C(8) | 8411(2) | 4588(1) | 2367(1) | 33(1) |
| C(9) | 8677(2) | 4876(1) | 3466(1) | 35(1) |
| C(10) | 8024(2) | 1447(1) | 4893(1) | 33(1) |
| C(11) | 7520(1) | 470(1) | 4595(1) | 30(1) |
| C(13) | 7903(1) | 665(1) | 2303(1) | 28(1) |
| C(14) | 7125(1) | 1028(1) | 1153(1) | 28(1) |
| C(15) | 5722(2) | 1719(1) | 1156(1) | 33(1) |
| C(16) | 5050(2) | 2040(2) | 48(1) | 36(1) |
| C(17) | 5746(2) | 1674(1) | −1079(1) | 33(1) |
| C(18) | 7135(2) | 959(2) | −1058(1) | 38(1) |
| C(19) | 7826(2) | 641(2) | 36(1) | 34(1) |
| C(20) | 5019(2) | 2037(2) | −2293(2) | 44(1) |
| C(21) | 7391(1) | −926(1) | 5434(1) | 30(1) |
| C(22) | 6461(2) | −1750(2) | 5198(1) | 35(1) |
| C(23) | 6324(2) | −3060(2) | 5988(2) | 40(1) |
| C(24) | 7107(2) | −3612(2) | 7038(2) | 39(1) |
| C(25) | 8040(2) | −2785(1) | 7265(1) | 35(1) |
| C(26) | 8182(2) | −1471(1) | 6487(1) | 32(1) |
| C(27) | 6945(2) | −5037(2) | 7895(2) | 56(1) |

**Table S3.** Bond lengths [Å] and angles [°] for **3a**.

| O(12)-C(13) | 1.3637(15) |
| --- | --- |
| O(12)-C(11) | 1.3998(16) |
| O(13)-C(13) | 1.2065(16) |
| O(1)-C(2) | 1.247(14) |
| O(1)-C(8) | 1.433(11) |
| N(3)-C(9) | 1.334(17) |
| N(3)-C(2) | 1.438(18) |
| N(1)-C(8) | 1.338(16) |
| N(1)-C(2) | 1.434(18) |
| O(3)-C(2) | 1.256(15) |
| O(3)-C(9) | 1.434(12) |
| C(2)-C(10) | 1.439(2) |
| C(4)-C(5) | 1.387(2) |
| C(4)-C(9) | 1.388(2) |
| C(4)-H(4) | 0.9500 |
| C(5)-C(6) | 1.395(2) |
| C(5)-H(5) | 0.9500 |
| C(6)-C(7) | 1.390(2) |
| C(6)-H(6) | 0.9500 |
| C(7)-C(8) | 1.378(2) |
| C(7)-H(7) | 0.9500 |
| C(8)-C(9) | 1.389(2) |
| C(10)-C(11) | 1.3356(19) |
| C(10)-H(10) | 0.9500 |
| C(11)-C(21) | 1.467(2) |
| C(13)-C(14) | 1.4771(18) |
| C(14)-C(15) | 1.3878(19) |
| C(14)-C(19) | 1.3938(18) |
| C(15)-C(16) | 1.382(2) |
| C(15)-H(15) | 0.9500 |
| C(16)-C(17) | 1.3903(19) |
| C(16)-H(16) | 0.9500 |
| C(17)-C(18) | 1.389(2) |
| C(17)-C(20) | 1.5095(19) |
| C(18)-C(19) | 1.380(2) |
| C(18)-H(18) | 0.9500 |
| C(19)-H(19) | 0.9500 |
| C(20)-H(20A) | 0.9800 |
| C(20)-H(20B) | 0.9800 |
| C(20)-H(20C) | 0.9800 |
| C(20)-H(20D) | 0.9800 |
| C(20)-H(20E) | 0.9800 |
| C(20)-H(20F) | 0.9800 |
| C(21)-C(22) | 1.4000(19) |
| C(21)-C(26) | 1.4002(19) |

**Table S3.** *Cont.*

| C(22)-C(23) | 1.379(2) |
| --- | --- |
| C(22)-H(22) | 0.9500 |
| C(23)-C(24) | 1.393(2) |
| C(23)-H(23) | 0.9500 |
| C(24)-C(25) | 1.398(2) |
| C(24)-C(27) | 1.502(2) |
| C(25)-C(26) | 1.380(2) |
| C(25)-H(25) | 0.9500 |
| C(26)-H(26) | 0.9500 |
| C(27)-H(27A) | 0.9800 |
| C(27)-H(27B) | 0.9800 |
| C(27)-H(27C) | 0.9800 |
| C(27)-H(27D) | 0.9800 |
| C(27)-H(27E) | 0.9800 |
| C(27)-H(27F) | 0.9800 |
| C(13)-O(12)-C(11) | 117.29(10) |
| C(2)-O(1)-C(8) | 106.7(8) |
| C(9)-N(3)-C(2) | 101.9(12) |
| C(8)-N(1)-C(2) | 101.8(11) |
| C(2)-O(3)-C(9) | 106.2(10) |
| O(3)-C(2)-N(1) | 114.8(6) |
| O(1)-C(2)-N(3) | 114.4(6) |
| O(1)-C(2)-C(10) | 124.1(5) |
| O(3)-C(2)-C(10) | 118.6(6) |
| N(1)-C(2)-C(10) | 126.4(7) |
| N(3)-C(2)-C(10) | 121.5(7) |
| C(5)-C(4)-C(9) | 116.16(14) |
| C(5)-C(4)-H(4) | 121.9 |
| C(9)-C(4)-H(4) | 121.9 |
| C(4)-C(5)-C(6) | 121.92(14) |
| C(4)-C(5)-H(5) | 119.0 |
| C(6)-C(5)-H(5) | 119.0 |
| C(7)-C(6)-C(5) | 121.52(15) |
| C(7)-C(6)-H(6) | 119.2 |
| C(5)-C(6)-H(6) | 119.2 |
| C(8)-C(7)-C(6) | 116.36(14) |
| C(8)-C(7)-H(7) | 121.8 |
| C(6)-C(7)-H(7) | 121.8 |
| N(1)-C(8)-C(7) | 125.8(8) |
| N(1)-C(8)-C(9) | 111.9(8) |
| C(7)-C(8)-C(9) | 122.25(13) |
| C(7)-C(8)-O(1) | 132.7(6) |
| C(9)-C(8)-O(1) | 104.9(6) |
| N(3)-C(9)-C(4) | 126.5(8) |
| N(3)-C(9)-C(8) | 111.7(8) |

**Table S3.** *Cont.*

| C(4)-C(9)-C(8) | 121.77(14) |
| --- | --- |
| C(4)-C(9)-O(3) | 132.9(6) |
| C(8)-C(9)-O(3) | 105.1(6) |
| C(11)-C(10)-C(2) | 126.44(13) |
| C(11)-C(10)-H(10) | 116.8 |
| C(2)-C(10)-H(10) | 116.8 |
| C(10)-C(11)-O(12) | 118.69(13) |
| C(10)-C(11)-C(21) | 126.51(13) |
| O(12)-C(11)-C(21) | 114.59(11) |
| O(13)-C(13)-O(12) | 122.44(12) |
| O(13)-C(13)-C(14) | 126.35(12) |
| O(12)-C(13)-C(14) | 111.20(11) |
| C(15)-C(14)-C(19) | 119.19(12) |
| C(15)-C(14)-C(13) | 122.40(11) |
| C(19)-C(14)-C(13) | 118.40(12) |
| C(16)-C(15)-C(14) | 120.00(12) |
| C(16)-C(15)-H(15) | 120.0 |
| C(14)-C(15)-H(15) | 120.0 |
| C(15)-C(16)-C(17) | 121.32(13) |
| C(15)-C(16)-H(16) | 119.3 |
| C(17)-C(16)-H(16) | 119.3 |
| C(18)-C(17)-C(16) | 118.12(13) |
| C(18)-C(17)-C(20) | 120.57(13) |
| C(16)-C(17)-C(20) | 121.31(14) |
| C(19)-C(18)-C(17) | 121.19(12) |
| C(19)-C(18)-H(18) | 119.4 |
| C(17)-C(18)-H(18) | 119.4 |
| C(18)-C(19)-C(14) | 120.15(13) |
| C(18)-C(19)-H(19) | 119.9 |
| C(14)-C(19)-H(19) | 119.9 |
| C(17)-C(20)-H(20A) | 109.5 |
| C(17)-C(20)-H(20B) | 109.5 |
| H(20A)-C(20)-H(20B) | 109.5 |
| C(17)-C(20)-H(20C) | 109.5 |
| H(20A)-C(20)-H(20C) | 109.5 |
| H(20B)-C(20)-H(20C) | 109.5 |
| C(17)-C(20)-H(20D) | 109.5 |
| H(20A)-C(20)-H(20D) | 141.1 |
| H(20B)-C(20)-H(20D) | 56.3 |
| H(20C)-C(20)-H(20D) | 56.3 |
| C(17)-C(20)-H(20E) | 109.5 |
| H(20A)-C(20)-H(20E) | 56.3 |
| H(20B)-C(20)-H(20E) | 141.1 |
| H(20C)-C(20)-H(20E) | 56.3 |
| H(20D)-C(20)-H(20E) | 109.5 |

**Table S3.** *Cont.*

| C(17)-C(20)-H(20F) | 109.5 |
| --- | --- |
| H(20A)-C(20)-H(20F) | 56.3 |
| H(20B)-C(20)-H(20F) | 56.3 |
| H(20C)-C(20)-H(20F) | 141.1 |
| H(20D)-C(20)-H(20F) | 109.5 |
| H(20E)-C(20)-H(20F) | 109.5 |
| C(22)-C(21)-C(26) | 118.10(13) |
| C(22)-C(21)-C(11) | 120.35(13) |
| C(26)-C(21)-C(11) | 121.55(12) |
| C(23)-C(22)-C(21) | 120.76(14) |
| C(23)-C(22)-H(22) | 119.6 |
| C(21)-C(22)-H(22) | 119.6 |
| C(22)-C(23)-C(24) | 121.54(13) |
| C(22)-C(23)-H(23) | 119.2 |
| C(24)-C(23)-H(23) | 119.2 |
| C(23)-C(24)-C(25) | 117.48(14) |
| C(23)-C(24)-C(27) | 121.11(14) |
| C(25)-C(24)-C(27) | 121.41(15) |
| C(26)-C(25)-C(24) | 121.62(14) |
| C(26)-C(25)-H(25) | 119.2 |
| C(24)-C(25)-H(25) | 119.2 |
| C(25)-C(26)-C(21) | 120.50(13) |
| C(25)-C(26)-H(26) | 119.8 |
| C(21)-C(26)-H(26) | 119.8 |
| C(24)-C(27)-H(27A) | 109.5 |
| C(24)-C(27)-H(27B) | 109.5 |
| H(27A)-C(27)-H(27B) | 109.5 |
| C(24)-C(27)-H(27C) | 109.5 |
| H(27A)-C(27)-H(27C) | 109.5 |
| H(27B)-C(27)-H(27C) | 109.5 |
| C(24)-C(27)-H(27D) | 109.5 |
| H(27A)-C(27)-H(27D) | 141.1 |
| H(27B)-C(27)-H(27D) | 56.3 |
| H(27C)-C(27)-H(27D) | 56.3 |
| C(24)-C(27)-H(27E) | 109.5 |
| H(27A)-C(27)-H(27E) | 56.3 |
| H(27B)-C(27)-H(27E) | 141.1 |
| H(27C)-C(27)-H(27E) | 56.3 |
| H(27D)-C(27)-H(27E) | 109.5 |
| C(24)-C(27)-H(27F) | 109.5 |
| H(27A)-C(27)-H(27F) | 56.3 |
| H(27B)-C(27)-H(27F) | 56.3 |
| H(27C)-C(27)-H(27F) | 141.1 |
| H(27D)-C(27)-H(27F) | 109.5 |
| H(27E)-C(27)-H(27F) | 109.5 |

**Table S4.** Anisotropic displacement parameters (Å^2^ × 10^3^) for **3a**. The anisotropic displacement factor exponent takes the form: −2π^2^[ h^2^a*^2^U^11^ + ... + 2 h k a* b* U^12^].

|  | **U^11^** | **U^22^** | **U^33^** | **U^23^** | **U^13^** | **U^12^** |
| --- | --- | --- | --- | --- | --- | --- |
| O(12) | 29(1) | 46(1) | 23(1) | −12(1) | −7(1) | 2(1) |
| O(13) | 30(1) | 43(1) | 33(1) | −18(1) | −8(1) | 3(1) |
| O(1) | 41(2) | 23(2) | 27(4) | −6(2) | −9(2) | −1(2) |
| N(3) | 41(3) | 33(4) | 41(3) | −20(2) | −10(2) | 0(3) |
| N(1) | 38(3) | 42(4) | 22(3) | −4(2) | −7(2) | 3(2) |
| O(3) | 41(3) | 26(3) | 30(2) | −10(2) | −6(2) | 1(3) |
| C(2) | 34(1) | 40(1) | 41(1) | −9(1) | −5(1) | −1(1) |
| C(4) | 40(1) | 44(1) | 44(1) | −21(1) | −10(1) | −1(1) |
| C(5) | 41(1) | 33(1) | 52(1) | −14(1) | −8(1) | 1(1) |
| C(6) | 45(1) | 37(1) | 41(1) | −7(1) | −8(1) | 3(1) |
| C(7) | 45(1) | 40(1) | 33(1) | −12(1) | −9(1) | 3(1) |
| C(8) | 34(1) | 32(1) | 34(1) | −13(1) | −6(1) | 2(1) |
| C(9) | 34(1) | 36(1) | 33(1) | −11(1) | −5(1) | 1(1) |
| C(10) | 36(1) | 39(1) | 25(1) | −9(1) | −6(1) | −2(1) |
| C(11) | 28(1) | 41(1) | 22(1) | −12(1) | −4(1) | 0(1) |
| C(13) | 30(1) | 28(1) | 27(1) | −11(1) | −4(1) | −2(1) |
| C(14) | 30(1) | 29(1) | 25(1) | −9(1) | −4(1) | −5(1) |
| C(15) | 34(1) | 39(1) | 28(1) | −14(1) | −5(1) | 1(1) |
| C(16) | 33(1) | 44(1) | 32(1) | −11(1) | −9(1) | 3(1) |
| C(17) | 34(1) | 40(1) | 26(1) | −8(1) | −7(1) | −10(1) |
| C(18) | 38(1) | 51(1) | 27(1) | −18(1) | −3(1) | −5(1) |
| C(19) | 30(1) | 43(1) | 31(1) | −16(1) | −4(1) | 0(1) |
| C(20) | 42(1) | 63(1) | 31(1) | −13(1) | −11(1) | −11(1) |
| C(21) | 28(1) | 37(1) | 25(1) | −13(1) | −3(1) | 0(1) |
| C(22) | 35(1) | 46(1) | 29(1) | −16(1) | −7(1) | −3(1) |
| C(23) | 40(1) | 43(1) | 39(1) | −19(1) | −3(1) | −8(1) |
| C(24) | 40(1) | 38(1) | 38(1) | −14(1) | −1(1) | −1(1) |
| C(25) | 33(1) | 39(1) | 33(1) | −11(1) | −7(1) | 4(1) |
| C(26) | 29(1) | 39(1) | 30(1) | −14(1) | −5(1) | −1(1) |
| C(27) | 61(1) | 41(1) | 60(1) | −9(1) | −9(1) | −6(1) |

**Table S5.** Hydrogen coordinates (×10^4^) and isotropic displacement parameters (Å^2^ × 10^3^) for **3a**.

|  | **x** | **y** | **z** | **U(eq)** |
| --- | --- | --- | --- | --- |
| H(4) | 9246 | 6332 | 4127 | 49 |
| H(5) | 9499 | 7937 | 2006 | 50 |
| H(6) | 9077 | 7442 | 169 | 51 |
| H(7) | 8350 | 5317 | 373 | 47 |
| H(10) | 8305 | 1222 | 5743 | 40 |
| H(15) | 5223 | 1971 | 1919 | 40 |
| H(16) | 4093 | 2520 | 57 | 44 |
| H(18) | 7619 | 683 | −1811 | 45 |
| H(19) | 8781 | 158 | 27 | 41 |
| H(20A) | 4052 | 2539 | −2134 | 66 |
| H(20B) | 4843 | 1221 | −2466 | 66 |
| H(20C) | 5690 | 2586 | −3069 | 66 |
| H(20D) | 5671 | 1692 | −2979 | 66 |
| H(20E) | 4880 | 3010 | −2646 | 66 |
| H(20F) | 4033 | 1644 | −2044 | 66 |
| H(22) | 5918 | −1404 | 4486 | 42 |
| H(23) | 5681 | −3599 | 5812 | 47 |
| H(25) | 8589 | −3137 | 7973 | 42 |
| H(26) | 8822 | −933 | 6667 | 38 |
| H(27A) | 7576 | −5245 | 8576 | 83 |
| H(27B) | 7268 | −5628 | 7338 | 83 |
| H(27C) | 5884 | −5169 | 8331 | 83 |
| H(27D) | 6243 | −5450 | 7587 | 83 |
| H(27E) | 6550 | −5067 | 8826 | 83 |
| H(27F) | 7935 | −5526 | 7832 | 83 |

**Table S6.** Torsion angles [°] for **3a**.

| C(8)-O(1)-C(2)-N(3) | 3.3(17) |
| --- | --- |
| C(8)-O(1)-C(2)-C(10) | −176.7(4) |
| C(9)-O(3)-C(2)-N(1) | 3.0(17) |
| C(9)-O(3)-C(2)-C(10) | 178.4(6) |
| C(8)-N(1)-C(2)-O(3) | −0.8(18) |
| C(8)-N(1)-C(2)-C(10) | −175.7(5) |
| C(9)-N(3)-C(2)-O(1) | −6.0(19) |
| C(9)-N(3)-C(2)-C(10) | 174.0(7) |
| C(9)-C(4)-C(5)-C(6) | 0.5(2) |
| C(4)-C(5)-C(6)-C(7) | 0.4(2) |
| C(5)-C(6)-C(7)-C(8) | −0.8(2) |
| C(2)-N(1)-C(8)-C(7) | 176.5(4) |
| C(2)-N(1)-C(8)-C(9) | −1.9(15) |
| C(2)-N(1)-C(8)-O(1) | 11(12) |
| C(6)-C(7)-C(8)-N(1) | −177.9(12) |
| C(6)-C(7)-C(8)-C(9) | 0.4(2) |
| C(6)-C(7)-C(8)-O(1) | −175.4(9) |
| C(2)-O(1)-C(8)-N(1) | −167(14) |
| C(2)-O(1)-C(8)-C(7) | 176.9(4) |
| C(2)-O(1)-C(8)-C(9) | 0.6(12) |
| C(2)-N(3)-C(9)-C(4) | −175.9(5) |
| C(2)-N(3)-C(9)-C(8) | 6.1(16) |
| C(2)-N(3)-C(9)-O(3) | −38(10) |
| C(5)-C(4)-C(9)-N(3) | −178.8(13) |
| C(5)-C(4)-C(9)-C(8) | −1.0(2) |
| C(5)-C(4)-C(9)-O(3) | 172.9(10) |
| N(1)-C(8)-C(9)-N(3) | −2.9(15) |
| C(7)-C(8)-C(9)-N(3) | 178.6(11) |
| O(1)-C(8)-C(9)-N(3) | −4.6(13) |
| N(1)-C(8)-C(9)-C(4) | 179.0(10) |
| C(7)-C(8)-C(9)-C(4) | 0.5(2) |
| O(1)-C(8)-C(9)-C(4) | 177.3(7) |
| N(1)-C(8)-C(9)-O(3) | 3.7(13) |
| C(7)-C(8)-C(9)-O(3) | −174.8(8) |
| O(1)-C(8)-C(9)-O(3) | 2.0(10) |
| C(2)-O(3)-C(9)-N(3) | 134(11) |
| C(2)-O(3)-C(9)-C(4) | −178.5(4) |
| C(2)-O(3)-C(9)-C(8) | −3.9(13) |
| O(1)-C(2)-C(10)-C(11) | −12.4(10) |
| O(3)-C(2)-C(10)-C(11) | 174.6(10) |
| N(1)-C(2)-C(10)-C(11) | −10.6(11) |
| N(3)-C(2)-C(10)-C(11) | 167.6(11) |
| C(2)-C(10)-C(11)-O(12) | −7.2(2) |
| C(2)-C(10)-C(11)-C(21) | 178.35(13) |
| C(13)-O(12)-C(11)-C(10) | 93.59(15) |

**Table S6.** *Cont.*

| C(13)-O(12)-C(11)-C(21) | −91.31(13) |
| --- | --- |
| C(11)-O(12)-C(13)-O(13) | 1.29(18) |
| C(11)-O(12)-C(13)-C(14) | 179.76(11) |
| O(13)-C(13)-C(14)-C(15) | −166.33(13) |
| O(12)-C(13)-C(14)-C(15) | 15.27(17) |
| O(13)-C(13)-C(14)-C(19) | 14.6(2) |
| O(12)-C(13)-C(14)-C(19) | −163.82(11) |
| C(19)-C(14)-C(15)-C(16) | −1.4(2) |
| C(13)-C(14)-C(15)-C(16) | 179.57(13) |
| C(14)-C(15)-C(16)-C(17) | 0.6(2) |
| C(15)-C(16)-C(17)-C(18) | 0.8(2) |
| C(15)-C(16)-C(17)-C(20) | −179.41(14) |
| C(16)-C(17)-C(18)-C(19) | −1.4(2) |
| C(20)-C(17)-C(18)-C(19) | 178.83(14) |
| C(17)-C(18)-C(19)-C(14) | 0.6(2) |
| C(15)-C(14)-C(19)-C(18) | 0.8(2) |
| C(13)-C(14)-C(19)-C(18) | 179.90(12) |
| C(10)-C(11)-C(21)-C(22) | 161.47(13) |
| O(12)-C(11)-C(21)-C(22) | −13.17(17) |
| C(10)-C(11)-C(21)-C(26) | −18.4(2) |
| O(12)-C(11)-C(21)-C(26) | 166.96(11) |
| C(26)-C(21)-C(22)-C(23) | 0.38(19) |
| C(11)-C(21)-C(22)-C(23) | −179.49(12) |
| C(21)-C(22)-C(23)-C(24) | −0.4(2) |
| C(22)-C(23)-C(24)-C(25) | 0.1(2) |
| C(22)-C(23)-C(24)-C(27) | 179.76(14) |
| C(23)-C(24)-C(25)-C(26) | 0.2(2) |
| C(27)-C(24)-C(25)-C(26) | −179.43(14) |
| C(24)-C(25)-C(26)-C(21) | −0.2(2) |
| C(22)-C(21)-C(26)-C(25) | −0.06(19) |
| C(11)-C(21)-C(26)-C(25) | 179.81(12) |

**Table S7.** Crystal data and structure refinement for **4a**.

| CCDC code | 922097 |  |
| --- | --- | --- |
| Empirical formula | C_24_H_19_NO_3_ |  |
| Formula weight | 369.40 |  |
| Temperature | 123(2) K |  |
| Wavelength | 1.54184 Å |  |
| Crystal system | Triclinic |  |
| Space group | *P*-1 |  |
| Unit cell dimensions | a = 8.2713(3) Å | α = 108.347(3)° |
|  | b = 11.0619(4) Å | β = 104.143(3)° |
|  | c = 11.1918(4) Å | γ = 93.746(3)° |
| Volume | 931.18(6) Å^3^ |  |
| Z | 2 |  |
| Density (calculated) | 1.317 Mg/m^3^ |  |
| Absorption coefficient | 0.699 mm^−1^ |  |
| F(000) | 388 |  |
| Crystal size | 0.20 × 0.17 × 0.12 mm^3^ |  |
| Theta range for data collection | 4.26 to 72.48° |  |
| Index ranges | −10 ≤ h ≤ 10, −13 ≤ k ≤ 13, −13 ≤ l ≤ 13 |  |
| Reflections collected | 15894 |  |
| Independent reflections | 3677 [R(int) = 0.0273] |  |
| Completeness to theta = 72.48° | 99.4% |  |
| Absorption correction | Semi-empirical from equivalents |  |
| Max. and min. transmission | 0.9208 and 0.8728 |  |
| Refinement method | Full-matrix least-squares on F^2^ |  |
| Data / restraints / parameters | 3677 / 3 / 253 |  |
| Goodness-of-fit on F^2^ | 1.022 |  |
| Final R indices [I > 2sigma(I)] | R1 = 0.0383, wR2 = 0.0988 |  |
| R indices (all data) | R1 = 0.0428, wR2 = 0.1027 |  |
| Largest diff. peak and hole | 0.241 and −0.234 e.Å^−3^ |  |

**Table S8.** Atomic coordinates (×10^4^) and equivalent isotropic displacement parameters
(Å^2^ × 10^3^) for **4a**. U(eq) is defined as one third of the trace of the orthogonalized U^ij^ tensor.

|  | **x** | **y** | **z** | **U(eq)** |
| --- | --- | --- | --- | --- |
| O(12) | 1464(1) | 830(1) | 3795(1) | 29(1) |
| O(13) | 3865(1) | 2228(1) | 4386(1) | 36(1) |
| O(1) | 3088(1) | 329(1) | 7489(1) | 34(1) |
| N(3) | 1749(1) | 1664(1) | 6562(1) | 30(1) |
| C(2) | 2520(2) | 668(1) | 6385(1) | 31(1) |
| C(4) | 1135(2) | 3062(1) | 8640(1) | 37(1) |
| C(5) | 1379(2) | 3194(1) | 9947(1) | 41(1) |
| C(6) | 2228(2) | 2354(2) | 10486(1) | 42(1) |
| C(7) | 2862(2) | 1342(1) | 9743(1) | 40(1) |
| C(8) | 2597(2) | 1227(1) | 8444(1) | 32(1) |
| C(9) | 1769(2) | 2053(1) | 7885(1) | 30(1) |
| C(10) | 2875(2) | −180(1) | 5238(1) | 29(1) |
| C(11) | 2413(1) | −117(1) | 4028(1) | 27(1) |
| C(13) | 2348(2) | 2005(1) | 4031(1) | 28(1) |
| C(14) | 1217(2) | 2929(1) | 3760(1) | 27(1) |
| C(15) | −529(2) | 2621(1) | 3443(1) | 29(1) |
| C(16) | −1571(2) | 3501(1) | 3188(1) | 31(1) |
| C(17) | −821(2) | 4697(1) | 3255(1) | 33(1) |
| C(18) | 921(2) | 5005(1) | 3564(1) | 34(1) |
| C(19) | 1950(2) | 4126(1) | 3822(1) | 31(1) |
| C(20) | −3456(2) | 3173(1) | 2889(2) | 39(1) |
| C(21) | 2720(2) | −1018(1) | 2850(1) | 28(1) |
| C(22) | 3512(2) | −2084(1) | 2923(1) | 30(1) |
| C(23) | 3797(2) | −2961(1) | 1821(1) | 33(1) |
| C(24) | 3274(2) | −2766(1) | 627(1) | 37(1) |
| C(25) | 2495(2) | −1713(1) | 538(1) | 39(1) |
| C(26) | 2218(2) | −839(1) | 1644(1) | 33(1) |
| C(27) | 4674(2) | −4089(1) | 1951(2) | 40(1) |

**Table S9.** Bond lengths [Å] and angles [°] for **4a**.

| O(12)-C(13) | 1.3636(15) |
| --- | --- |
| O(12)-C(11) | 1.3999(14) |
| O(13)-C(13) | 1.2036(15) |
| O(1)-C(8) | 1.3757(15) |
| O(1)-C(2) | 1.3845(15) |
| N(3)-C(2) | 1.2953(16) |
| N(3)-C(9) | 1.4009(16) |
| C(2)-C(10) | 1.4413(17) |
| C(4)-C(5) | 1.384(2) |
| C(4)-C(9) | 1.3914(18) |
| C(4)-H(4) | 0.9500 |
| C(5)-C(6) | 1.397(2) |
| C(5)-H(5) | 0.9500 |
| C(6)-C(7) | 1.386(2) |
| C(6)-H(6) | 0.9500 |
| C(7)-C(8) | 1.3791(18) |
| C(7)-H(7) | 0.9500 |
| C(8)-C(9) | 1.3882(18) |
| C(10)-C(11) | 1.3398(18) |
| C(10)-H(10) | 0.9500 |
| C(11)-C(21) | 1.4709(17) |
| C(13)-C(14) | 1.4805(16) |
| C(14)-C(15) | 1.3915(17) |
| C(14)-C(19) | 1.3936(18) |
| C(15)-C(16) | 1.3913(17) |
| C(15)-H(15) | 0.9500 |
| C(16)-C(17) | 1.3957(19) |
| C(16)-C(20) | 1.5063(18) |
| C(17)-C(18) | 1.3890(19) |
| C(17)-H(17) | 0.9500 |
| C(18)-C(19) | 1.3848(18) |
| C(18)-H(18) | 0.9500 |
| C(19)-H(19) | 0.9500 |
| C(20)-H(20A) | 0.9800 |
| C(20)-H(20B) | 0.9800 |
| C(20)-H(20C) | 0.9800 |
| C(20)-H(20D) | 0.9800 |
| C(20)-H(20E) | 0.9800 |
| C(20)-H(20F) | 0.9800 |
| C(21)-C(26) | 1.3930(18) |
| C(21)-C(22) | 1.4011(17) |
| C(22)-C(23) | 1.3916(18) |
| C(22)-H(22) | 0.9500 |
| C(23)-C(24) | 1.389(2) |
| C(23)-C(27) | 1.5115(18) |

**Table S9.** *Cont.*

| C(24)-C(25) | 1.387(2) |
| --- | --- |
| C(24)-H(24) | 0.9500 |
| C(25)-C(26) | 1.3901(19) |
| C(25)-H(25) | 0.9500 |
| C(26)-H(26) | 0.9500 |
| C(27)-H(27A) | 0.9800 |
| C(27)-H(27B) | 0.9800 |
| C(27)-H(27C) | 0.9800 |
| C(27)-H(27D) | 0.9800 |
| C(27)-H(27E) | 0.9800 |
| C(27)-H(27F) | 0.9800 |
| C(13)-O(12)-C(11) | 116.54(9) |
| C(8)-O(1)-C(2) | 103.87(10) |
| C(2)-N(3)-C(9) | 104.74(11) |
| N(3)-C(2)-O(1) | 114.85(11) |
| N(3)-C(2)-C(10) | 131.86(12) |
| O(1)-C(2)-C(10) | 113.28(11) |
| C(5)-C(4)-C(9) | 117.10(13) |
| C(5)-C(4)-H(4) | 121.5 |
| C(9)-C(4)-H(4) | 121.5 |
| C(4)-C(5)-C(6) | 121.55(13) |
| C(4)-C(5)-H(5) | 119.2 |
| C(6)-C(5)-H(5) | 119.2 |
| C(7)-C(6)-C(5) | 121.96(13) |
| C(7)-C(6)-H(6) | 119.0 |
| C(5)-C(6)-H(6) | 119.0 |
| C(8)-C(7)-C(6) | 115.40(13) |
| C(8)-C(7)-H(7) | 122.3 |
| C(6)-C(7)-H(7) | 122.3 |
| O(1)-C(8)-C(7) | 128.11(13) |
| O(1)-C(8)-C(9) | 107.98(11) |
| C(7)-C(8)-C(9) | 123.90(13) |
| C(8)-C(9)-C(4) | 120.08(12) |
| C(8)-C(9)-N(3) | 108.55(11) |
| C(4)-C(9)-N(3) | 131.37(12) |
| C(11)-C(10)-C(2) | 126.26(12) |
| C(11)-C(10)-H(10) | 116.9 |
| C(2)-C(10)-H(10) | 116.9 |
| C(10)-C(11)-O(12) | 119.50(11) |
| C(10)-C(11)-C(21) | 125.93(11) |
| O(12)-C(11)-C(21) | 114.46(10) |
| O(13)-C(13)-O(12) | 122.79(11) |
| O(13)-C(13)-C(14) | 125.39(11) |
| O(12)-C(13)-C(14) | 111.79(10) |
| C(15)-C(14)-C(19) | 120.35(11) |

**Table S9.** *Cont.*

| C(15)-C(14)-C(13) | 121.60(11) |
| --- | --- |
| C(19)-C(14)-C(13) | 118.05(11) |
| C(16)-C(15)-C(14) | 120.75(12) |
| C(16)-C(15)-H(15) | 119.6 |
| C(14)-C(15)-H(15) | 119.6 |
| C(15)-C(16)-C(17) | 118.32(12) |
| C(15)-C(16)-C(20) | 120.21(12) |
| C(17)-C(16)-C(20) | 121.44(11) |
| C(18)-C(17)-C(16) | 121.09(12) |
| C(18)-C(17)-H(17) | 119.5 |
| C(16)-C(17)-H(17) | 119.5 |
| C(19)-C(18)-C(17) | 120.24(12) |
| C(19)-C(18)-H(18) | 119.9 |
| C(17)-C(18)-H(18) | 119.9 |
| C(18)-C(19)-C(14) | 119.25(12) |
| C(18)-C(19)-H(19) | 120.4 |
| C(14)-C(19)-H(19) | 120.4 |
| C(16)-C(20)-H(20A) | 109.5 |
| C(16)-C(20)-H(20B) | 109.5 |
| H(20A)-C(20)-H(20B) | 109.5 |
| C(16)-C(20)-H(20C) | 109.5 |
| H(20A)-C(20)-H(20C) | 109.5 |
| H(20B)-C(20)-H(20C) | 109.5 |
| C(16)-C(20)-H(20D) | 109.5 |
| H(20A)-C(20)-H(20D) | 141.1 |
| H(20B)-C(20)-H(20D) | 56.3 |
| H(20C)-C(20)-H(20D) | 56.3 |
| C(16)-C(20)-H(20E) | 109.5 |
| H(20A)-C(20)-H(20E) | 56.3 |
| H(20B)-C(20)-H(20E) | 141.1 |
| H(20C)-C(20)-H(20E) | 56.3 |
| H(20D)-C(20)-H(20E) | 109.5 |
| C(16)-C(20)-H(20F) | 109.5 |
| H(20A)-C(20)-H(20F) | 56.3 |
| H(20B)-C(20)-H(20F) | 56.3 |
| H(20C)-C(20)-H(20F) | 141.1 |
| H(20D)-C(20)-H(20F) | 109.5 |
| H(20E)-C(20)-H(20F) | 109.5 |
| C(26)-C(21)-C(22) | 118.71(12) |
| C(26)-C(21)-C(11) | 120.99(11) |
| C(22)-C(21)-C(11) | 120.30(11) |
| C(23)-C(22)-C(21) | 121.48(12) |
| C(23)-C(22)-H(22) | 119.3 |
| C(21)-C(22)-H(22) | 119.3 |
| C(24)-C(23)-C(22) | 118.70(12) |

**Table S9.** *Cont.*

| C(24)-C(23)-C(27) | 121.79(12) |
| --- | --- |
| C(22)-C(23)-C(27) | 119.51(12) |
| C(25)-C(24)-C(23) | 120.63(12) |
| C(25)-C(24)-H(24) | 119.7 |
| C(23)-C(24)-H(24) | 119.7 |
| C(24)-C(25)-C(26) | 120.37(13) |
| C(24)-C(25)-H(25) | 119.8 |
| C(26)-C(25)-H(25) | 119.8 |
| C(25)-C(26)-C(21) | 120.11(12) |
| C(25)-C(26)-H(26) | 119.9 |
| C(21)-C(26)-H(26) | 119.9 |
| C(23)-C(27)-H(27A) | 109.5 |
| C(23)-C(27)-H(27B) | 109.5 |
| H(27A)-C(27)-H(27B) | 109.5 |
| C(23)-C(27)-H(27C) | 109.5 |
| H(27A)-C(27)-H(27C) | 109.5 |
| H(27B)-C(27)-H(27C) | 109.5 |
| C(23)-C(27)-H(27D) | 109.5 |
| H(27A)-C(27)-H(27D) | 141.1 |
| H(27B)-C(27)-H(27D) | 56.3 |
| H(27C)-C(27)-H(27D) | 56.3 |
| C(23)-C(27)-H(27E) | 109.5 |
| H(27A)-C(27)-H(27E) | 56.3 |
| H(27B)-C(27)-H(27E) | 141.1 |
| H(27C)-C(27)-H(27E) | 56.3 |
| H(27D)-C(27)-H(27E) | 109.5 |
| C(23)-C(27)-H(27F) | 109.5 |
| H(27A)-C(27)-H(27F) | 56.3 |
| H(27B)-C(27)-H(27F) | 56.3 |
| H(27C)-C(27)-H(27F) | 141.1 |
| H(27D)-C(27)-H(27F) | 109.5 |
| H(27E)-C(27)-H(27F) | 109.5 |

**Table S10.** Anisotropic displacement parameters (Å^2^ × 10^3^) for **4a**. The anisotropic displacement factor exponent takes the form: −2π^2^[h^2^a*^2^U^11^ + ... + 2 h k a* b* U^12^].

|  | **U^11^** | **U^22^** | **U^33^** | **U^23^** | **U^13^** | **U^12^** |
| --- | --- | --- | --- | --- | --- | --- |
| O(12) | 25(1) | 26(1) | 35(1) | 10(1) | 9(1) | 7(1) |
| O(13) | 25(1) | 36(1) | 49(1) | 19(1) | 11(1) | 6(1) |
| O(1) | 37(1) | 32(1) | 34(1) | 12(1) | 12(1) | 5(1) |
| N(3) | 30(1) | 32(1) | 30(1) | 9(1) | 12(1) | 5(1) |
| C(2) | 27(1) | 31(1) | 32(1) | 10(1) | 9(1) | 1(1) |
| C(4) | 36(1) | 34(1) | 40(1) | 9(1) | 17(1) | 4(1) |
| C(5) | 42(1) | 40(1) | 39(1) | 3(1) | 19(1) | 0(1) |
| C(6) | 47(1) | 47(1) | 30(1) | 8(1) | 13(1) | −5(1) |
| C(7) | 45(1) | 41(1) | 33(1) | 15(1) | 10(1) | −1(1) |
| C(8) | 33(1) | 30(1) | 31(1) | 7(1) | 12(1) | −1(1) |
| C(9) | 29(1) | 30(1) | 29(1) | 7(1) | 11(1) | 0(1) |
| C(10) | 26(1) | 27(1) | 33(1) | 9(1) | 10(1) | 5(1) |
| C(11) | 22(1) | 25(1) | 35(1) | 10(1) | 9(1) | 5(1) |
| C(13) | 29(1) | 29(1) | 27(1) | 9(1) | 12(1) | 6(1) |
| C(14) | 29(1) | 27(1) | 26(1) | 8(1) | 11(1) | 7(1) |
| C(15) | 30(1) | 27(1) | 31(1) | 9(1) | 12(1) | 6(1) |
| C(16) | 30(1) | 34(1) | 31(1) | 12(1) | 13(1) | 10(1) |
| C(17) | 38(1) | 34(1) | 37(1) | 16(1) | 16(1) | 15(1) |
| C(18) | 39(1) | 30(1) | 39(1) | 15(1) | 16(1) | 7(1) |
| C(19) | 29(1) | 32(1) | 33(1) | 10(1) | 12(1) | 5(1) |
| C(20) | 30(1) | 43(1) | 51(1) | 21(1) | 14(1) | 12(1) |
| C(21) | 24(1) | 27(1) | 32(1) | 9(1) | 9(1) | 2(1) |
| C(22) | 25(1) | 27(1) | 37(1) | 9(1) | 9(1) | 4(1) |
| C(23) | 27(1) | 27(1) | 42(1) | 7(1) | 13(1) | 3(1) |
| C(24) | 40(1) | 33(1) | 37(1) | 4(1) | 18(1) | 5(1) |
| C(25) | 44(1) | 40(1) | 33(1) | 12(1) | 14(1) | 5(1) |
| C(26) | 33(1) | 30(1) | 35(1) | 11(1) | 10(1) | 6(1) |
| C(27) | 37(1) | 29(1) | 51(1) | 6(1) | 16(1) | 9(1) |

**Table S11.** Hydrogen coordinates (×10^4^) and isotropic displacement parameters (Å^2^ × 10^3^) for **4a**.

|  | **x** | **y** | **z** | **U(eq)** |
| --- | --- | --- | --- | --- |
| H(4) | 560 | 3635 | 8276 | 44 |
| H(5) | 958 | 3872 | 10489 | 50 |
| H(6) | 2375 | 2480 | 11388 | 51 |
| H(7) | 3440 | 768 | 10105 | 47 |
| H(10) | 3497 | −848 | 5350 | 34 |
| H(15) | −1015 | 1801 | 3399 | 34 |
| H(17) | −1513 | 5310 | 3086 | 40 |
| H(18) | 1408 | 5821 | 3598 | 41 |
| H(19) | 3142 | 4336 | 4039 | 37 |
| H(20A) | −3739 | 2297 | 2886 | 59 |
| H(20B) | −4001 | 3221 | 2026 | 59 |
| H(20C) | −3853 | 3786 | 3559 | 59 |
| H(20D) | −3989 | 3905 | 2762 | 59 |
| H(20E) | −3728 | 2982 | 3621 | 59 |
| H(20F) | −3876 | 2417 | 2089 | 59 |
| H(22) | 3864 | −2211 | 3743 | 36 |
| H(24) | 3451 | −3361 | −135 | 45 |
| H(25) | 2148 | −1588 | −283 | 46 |
| H(26) | 1687 | −118 | 1576 | 39 |
| H(27A) | 4778 | −4611 | 1094 | 60 |
| H(27B) | 4014 | −4618 | 2274 | 60 |
| H(27C) | 5800 | −3768 | 2571 | 60 |
| H(27D) | 4950 | −4054 | 2866 | 60 |
| H(27E) | 5715 | −4046 | 1685 | 60 |
| H(27F) | 3928 | −4897 | 1388 | 60 |

**Table S12.** Torsion angles [°] for **4a**.

| C(9)-N(3)-C(2)-O(1) | −0.26(14) |
| --- | --- |
| C(9)-N(3)-C(2)-C(10) | 178.37(13) |
| C(8)-O(1)-C(2)-N(3) | 0.16(14) |
| C(8)-O(1)-C(2)-C(10) | −178.73(10) |
| C(9)-C(4)-C(5)-C(6) | 0.2(2) |
| C(4)-C(5)-C(6)-C(7) | −0.4(2) |
| C(5)-C(6)-C(7)-C(8) | 0.0(2) |
| C(2)-O(1)-C(8)-C(7) | −179.25(13) |
| C(2)-O(1)-C(8)-C(9) | 0.02(13) |
| C(6)-C(7)-C(8)-O(1) | 179.63(13) |
| C(6)-C(7)-C(8)-C(9) | 0.5(2) |
| O(1)-C(8)-C(9)-C(4) | −179.96(11) |
| C(7)-C(8)-C(9)-C(4) | −0.7(2) |
| O(1)-C(8)-C(9)-N(3) | −0.17(14) |
| C(7)-C(8)-C(9)-N(3) | 179.14(12) |
| C(5)-C(4)-C(9)-C(8) | 0.30(19) |
| C(5)-C(4)-C(9)-N(3) | −179.44(13) |
| C(2)-N(3)-C(9)-C(8) | 0.26(13) |
| C(2)-N(3)-C(9)-C(4) | −179.98(14) |
| N(3)-C(2)-C(10)-C(11) | −1.6(2) |
| O(1)-C(2)-C(10)-C(11) | 177.04(11) |
| C(2)-C(10)-C(11)-O(12) | −1.30(19) |
| C(2)-C(10)-C(11)-C(21) | −177.32(11) |
| C(13)-O(12)-C(11)-C(10) | 85.78(14) |
| C(13)-O(12)-C(11)-C(21) | −97.76(12) |
| C(11)-O(12)-C(13)-O(13) | 2.02(17) |
| C(11)-O(12)-C(13)-C(14) | −179.66(9) |
| O(13)-C(13)-C(14)-C(15) | −175.35(12) |
| O(12)-C(13)-C(14)-C(15) | 6.37(16) |
| O(13)-C(13)-C(14)-C(19) | 5.09(18) |
| O(12)-C(13)-C(14)-C(19) | −173.19(10) |
| C(19)-C(14)-C(15)-C(16) | −0.31(18) |
| C(13)-C(14)-C(15)-C(16) | −179.86(11) |
| C(14)-C(15)-C(16)-C(17) | 0.24(18) |
| C(14)-C(15)-C(16)-C(20) | −178.01(12) |
| C(15)-C(16)-C(17)-C(18) | 0.13(19) |
| C(20)-C(16)-C(17)-C(18) | 178.37(12) |
| C(16)-C(17)-C(18)-C(19) | −0.4(2) |
| C(17)-C(18)-C(19)-C(14) | 0.38(19) |
| C(15)-C(14)-C(19)-C(18) | 0.00(18) |
| C(13)-C(14)-C(19)-C(18) | 179.56(11) |
| C(10)-C(11)-C(21)-C(26) | −178.32(12) |
| O(12)-C(11)-C(21)-C(26) | 5.49(17) |
| C(10)-C(11)-C(21)-C(22) | 2.27(19) |
| O(12)-C(11)-C(21)-C(22) | −173.92(10) |

**Table S12.** *Cont.*

| C(26)-C(21)-C(22)-C(23) | −0.33(18) |
| --- | --- |
| C(11)-C(21)-C(22)-C(23) | 179.09(11) |
| C(21)-C(22)-C(23)-C(24) | −0.15(19) |
| C(21)-C(22)-C(23)-C(27) | 179.32(11) |
| C(22)-C(23)-C(24)-C(25) | 0.5(2) |
| C(27)-C(23)-C(24)-C(25) | −178.97(13) |
| C(23)-C(24)-C(25)-C(26) | −0.3(2) |
| C(24)-C(25)-C(26)-C(21) | −0.2(2) |
| C(22)-C(21)-C(26)-C(25) | 0.48(19) |
| C(11)-C(21)-C(26)-C(25) | −178.94(12) |

© 2013 by the authors; licensee MDPI, Basel, Switzerland. This article is an open access article distributed under the terms and conditions of the Creative Commons Attribution license (http://creativecommons.org/licenses/by/3.0/).
